# Supplementary figures and images for: Daily high-frequency transcranial random noise stimulation (hf-tRNS) for sleep disturbances and cognitive dysfunction in patients with mild vascular cognitive impairments: A study protocol for a pilot randomized controlled trial
Source: PLoS One. 2024 Oct 23;19(10):e0309233. doi: 10.1371/journal.pone.0309233 (PMC11498659; doi:10.1371/journal.pone.0309233)

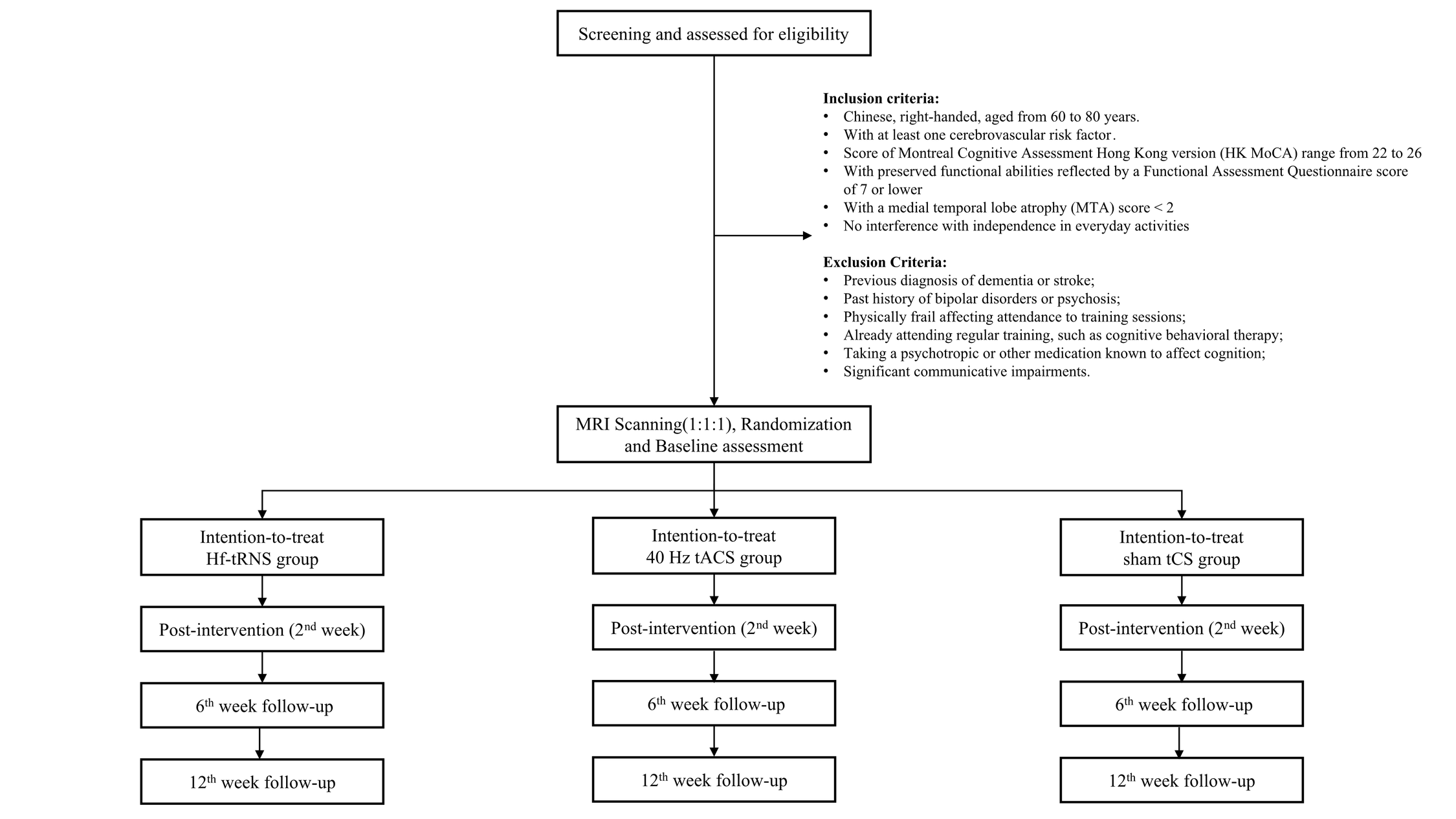

Supplement: S1 Fig — (TIF) [file pone.0309233.s002.tif]

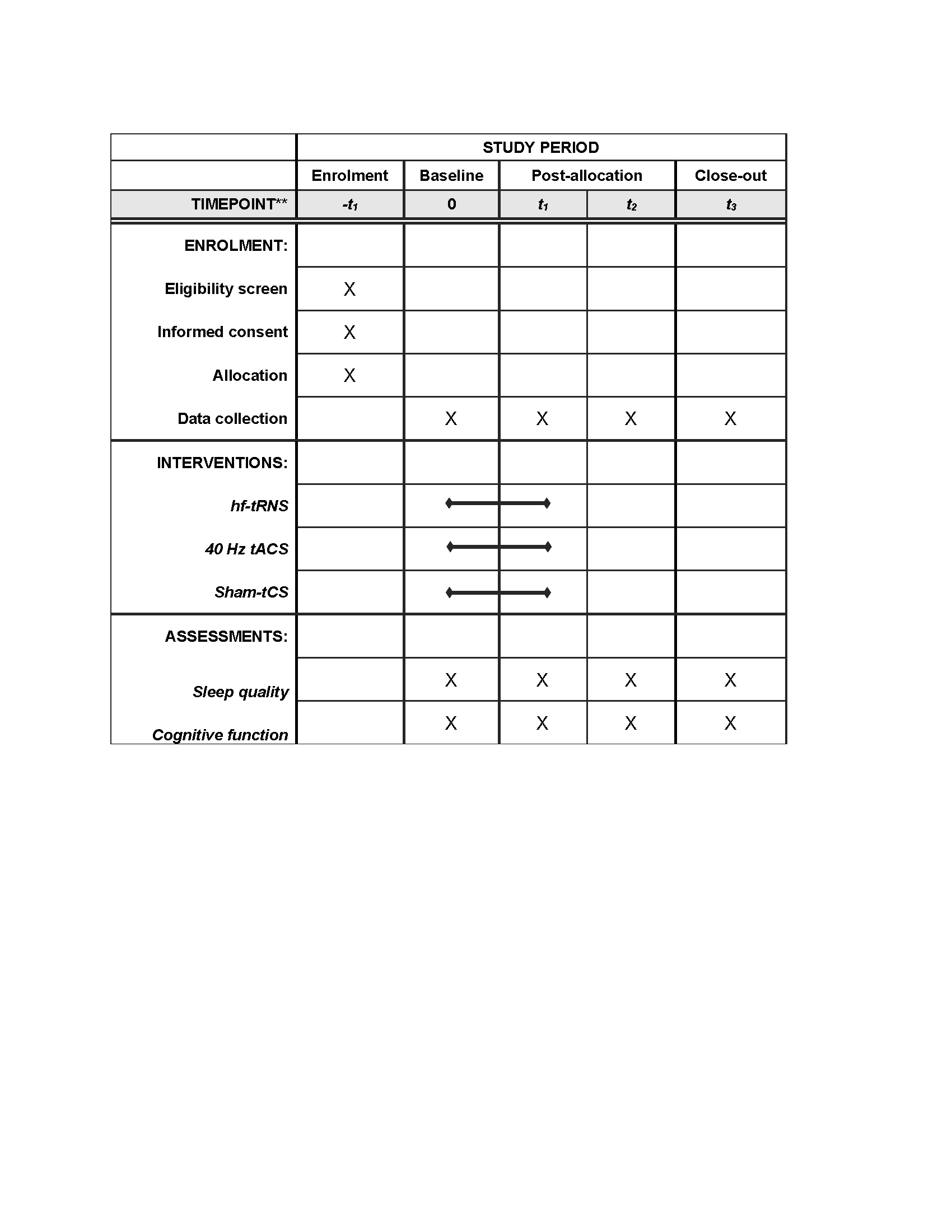

Supplement: S2 Fig — Abbreviations: hf-tRNS = High-frequency transcranial random noise stimulation; tACS = Transcranial alternating current stimulation; tCS = Transcranial current stimulation. (TIF) [file pone.0309233.s003.tif]
