# Supplementary material for: Daily high-frequency transcranial random noise stimulation (hf-tRNS) for sleep disturbances and cognitive dysfunction in patients with mild vascular cognitive impairments: A study protocol for a pilot randomized controlled trial
Source: PLoS One. 2024 Oct 23;19(10):e0309233. doi: 10.1371/journal.pone.0309233 (PMC11498659; doi:10.1371/journal.pone.0309233)
Supplement: S1 File — (PDF) [file pone.0309233.s004.pdf]

Research Protocol

A pilot randomized controlled trial of MRI-informed high-frequency transcranial random noise stimulation (hf-tRNS) for sleep disturbance and cognitive dysfunction in mild vascular cognitive impairment

Department of Psychiatry, The Chinese University of Hong Kong, Hong Kong SAR, China

Study objective (s) and significance

Study Objectives

1) To investigate the feasibility and safety of a 2-week high-frequency tRNS on sleep quality and cognition in mild vascular cognitive impairment patients.
2) To determine the sample size of a full-scale randomized clinical trial of tRNS in mild vascular cognitive impairment patients.
3) To evaluate the effects of oscillation-specific treatments on sleep quality, cognition and the function of glymphatic system at 1, 2 and 3 months after the treatments.

Significance

Sleep is a fundamental biological requirement for brain health. Poor sleep quality, as a modifiable risk factor, can jeopardize the cognition and healthy longevity (Svensson et al., 2021; Li et al., 2022). Of note, the status of sleep disturbances is increasingly considered to be an underlying cause of cerebrovascular diseases, such as stroke, vascular dementia (Garbarino et al., 2021). This is a slowly progressing preclinical condition that gradually lead to impaired cognition and reduced quality of life with ageing.

While sleep management in individuals at higher risks of developing stroke and vascular dementia is certainly important, growing evidence indicates that disruption of sleep slow oscillations (SSOs) can interfere with the strengths of specific synapses tagged as relevant neural circuits, adding to cognitive dysfunction in these individuals (Niethard et al., 2018; Paller et al., 2021). Notably, recent research suggests that slow oscillation activities can facilitate neurons in the process of cleaning toxic materials, removing them through intracranial cerebrospinal fluid (CSF) transport and glymphatic system (Hablitz & Nedergaard, 2021) and enhancing the sleep and cognitive functions correspondingly. This bidirectional relationship between brain oscillations, sleep quality and cognitive changes highlights the possibility to reverse this process through modulating the brain activities.

At present, non-pharmacological therapies for sleep disturbances are accepted as the first line of treatment in clinical guidelines (Peter-Derex et al., 2015). However, clinical trials examining the non-pharmacological therapies are very limited and current evidence

is lacking for therapies that are effective in the management of sleep disturbances in the individuals with higher risks of developing stroke and vascular dementia. As a form of novel non-invasive technology, transcranial current stimulation (tCS) shows increasing popularity because of its diverse modalities and positive effects on circadian rhythms and cognitive functions. For instance, transcranial alternating current stimulation (tACS) and transcranial random noise stimulation (tRNS), as the advanced modalities of tCS, could deliver the oscillation-specific waveforms of current that could modulate and monitor the brain activities in a more specific manner.

Work done by us

Our team has completed a clinical trial to compare the efficacy of 40Hz tACS on sleep quality and cognitive functions in senior adults with preclinical Alzheimer's disease (AD) (ClinicalTrials.gov Identifier: NCT05544201). In our preliminary results, we used the Pittsburgh Sleep Quality Index (PSQI) to evaluate the subjective sleep quality and found that repeated tACS has significant positive effects on sleep quality than sham tCS (PSQI score change: tACS group vs sham tCS: 6.01 vs 3.72, $p<0.001$). Although the promising results of tACS have been observed in preclinical AD patients, the potential mechanisms of oscillation-specific tCS on circadian rhythms and glymphatic system, and whether the oscillation-specific tCS can be used as a transdiagnostic non-pharmacological treatment in neurodegenerative diseases are still not known. Moreover, compared to the stimulation with fixed frequency, high-frequency tRNS can add electrical noise to cortical circuits to enhance neural processing and further induce prolonged physiological and excitability changes (Potok et al., 2022).

Collectively, there is insufficient evidence to support a large, full-scale randomized controlled trial (RCT) that involves the comparisons of the effects of oscillation-specific tCS on sleep quality and domain-specific cognitive functions, such as executive function and attention. There is also a lack of clinical data that would allow the estimation of the sample size or the efficacy and sustainability of oscillation-specific tCS for the full-scale RCT. Thus, this pilot RCT aims to test the safety and efficacy of oscillation-specific tCS (i.e., tRNS and tACS) for sleep disturbances and cognitive dysfunction in mild vascular cognitive impairment patients that also allow to determine the sample size of a full-scale

RCT. The findings of this study will provide valuable clinical evidence that can inform the effect size and personalized modeling of oscillation-specific tCS for age-related brain diseases. Furthermore, the dynamic changes of sleep quality, cognition and glymphatic system function observed in this pilot RCT will be helpful for in-depth understanding the relationship of "brain oscillations, sleep quality and cognition" and guiding the future studies of clinical neuroscience, brain diseases and sleep medicine.

Project duration

24 months

Research plan and methodology

Research Design

Randomized, double blind, controlled clinical trial

Methodology

Sources of data

Eligible participants will be recruited through our existing research cohort, i.e., Hong Kong Cohort of Abnormal Sleep in Ageing Population (HK-ASAP) (ClinicalTrials.gov Identifier: NCT06170073). The research team, including neuroscientists and psychiatrists, will identify the participants with mild vascular cognitive impairment (VCI). Potential participants will be invited to be screened by our trained research assistant to determine the eligibility and availability to participate in this pilot RCT. Both participants and their caregivers will be briefed about the study before a decision for informed consent.

Potential mild VCI patients will need to satisfy the following inclusion criteria:

1. Chinese, right-handed, aged from 60 to 80 years.

2. Score of Montreal Cognitive Assessment Hong Kong version (HK MoCA) range from 22 to 26 (Lu et al., 2019).

3. With impaired executive functions measured by HK MoCA.

4. With at least one cerebrovascular risk factor: (1) a history of hypertension defined as systolic blood pressure (BP) 140 mm Hg or higher, diastolic BP of 90 mm Hg or higher; or receiving antihypertensive medication; (2) comorbid with diabetes mellitus (DM) or hyperlipidemia or receiving related medication.

5. Based on structural MRI, with a medial temporal lobe atrophy (MTA) score < 2 (For the purpose of excluding the participants with prodromal Alzheimer's disease).

6. No interference with independence in everyday activities.

Exclusion criteria include:

1. Previous diagnosis of dementia or stroke.

2. Past history of bipolar disorders or other psychosis.

3. Physically frailty affecting attendance at training sessions.

4. Already attending regular training, such as cognitive behavioral therapy.

5. Taking a psychotropic medication or other medication known to affect cognition (e.g. anti-dementia medication).

6. Significant communicative impairments, including severe hearing loss and vision loss.

Neuroimaging

High-resolution structural MRI

All neuroimaging scans will be collected at the Prince of Wales Hospital using a 3.0 Tesla Siemens MAGNETOM Prisma MRI scanner using a 32-channel head coil. High resolution T1-weighted structural magnetic resonance imaging (MRI) scans will be acquired using a Magnetization Prepared RApid Gradient Echo (MPRAGE) sequence with the following parameters: axial acquisition with a 256×256×192 matrix, thickness = 1 mm, no gap, field of view (FOV) = 230 mm, repetition time (TR) = 2070 ms, echo time (TE) = 3.93 ms, flip angle = 15◦. The sequence yields high quality isotropic images with the voxel size of 1 mm ×1 mm × 1 mm (Saleh et al., 2004).

Diffusion tensor imaging (DTI)

For evaluating the function of glymphatic system, DTI will be conducted using spin-echo single-shot echo-planar pulse sequences with a total of 32 different diffusion directions: TR/TE = 11015/73.5 ms, slice thickness = 2.5 mm, acquisition matrix = 128 × 128, FOV = 224 × 224 mm^2, and b-value = 1000 s/mm^2. T2 star weighted angiography (SWAN) with TR/TE = 43.2/4.0 ms, slice thickness = 2 mm, acquisition matrix = 220 × 220 mm^2, and flip angle = 20° will be also used (Liang et al., 2023).

Randomization and masking

Participants will be randomly assigned 1:1:1 to one of three possible treatments: 1) high-definition (HD) tRNS, 2) 40 Hz HD-tACS, 3) sham HD-tCS. In order to ensure equally allocation across different treatment groups, prior to the enrollment, the randomization assignment will be generated using an online system (http://randomization.com/) by a statistician not involved in the study design. Assessment staff and participants will be blinded to the study design and group allocation.

Treatment schedule

The schedule of this pilot RCT is a 2-week course of treatment with 5 sessions per week, 20 minutes per session. All participants will receive a total of 10 sessions of treatment. The schedules for treatment are the same in three randomized groups.

Treatment strategies

Apparatus and settings

High-definition transcranial current stimulation (HD-tCS) is delivered by a battery driven direct current stimulator (DC-Stimulator Plus, NeuroConn, Ilmenau, Germany) through a central anodal electrode surrounded by four return cathodal electrodes. The base diameter of HD-tCS electrode is 2.4 cm. We place the center electrode (i.e., anodal) over left inferior parietal lobe (IPL) (i.e., P3 according to the international 10-20 EEG system) as in previous studies that investigated cognition and sleep (Murphy et al., 2009; Lai et al., 2023). To ensure the electrodes are secured in place, the locations of the electrodes will be measured and positioned based on individual structural MRI. The electrodes are fixed with conductive paste (Ten20®, Neurodiagnostic Electrode Paste, Weaver and Company,

Aurora, CO, USA). Participants are instructed to relax during the setting up of transcranial current stimulation.

Stimulation modalities

1. High-frequency transcranial random noise stimulation (hf-tRNS).

The stimulation parameters of tRNS include: 20 minutes at 101-640 Hz, 2 milliamps (van der Groen et al., 2022).

2. 40 Hz transcranial alternating current stimulation (tACS)

The stimulation parameters of tACS include: 20 minutes at 40 Hz, 2 milliamps (Lu et al., 2023).

3. Sham transcranial current stimulation (tCS)

In sham condition, the stimulation only lasts for 30 seconds with the electrodes left in place for a further 20 minutes. This procedure mimics the transient skin sensation of tingling induced by tRNS and tACS without producing any sustainable effects (Lu et al., 2019; 2023).

Group assignment

All the eligible participants will receive a total of 10 sessions of oscillation-specific tCS treatment. According to the modalities of tCS treatment, the participants will be randomly assigned to three groups:

1. hf-tRNS (101-640 Hz)

2. 40 Hz tACS

3. Sham tCS

Randomization

This is a double-blind, sham-controlled, randomized clinical trial. All the participants will be blinded to the group assignment (i.e., treatment). Independent research assistants who collect the inventory for sleep quality and cognitive functions will be blinded and will not participate in other outcome assessments.

Outcome assessments

Primary outcomes

1. Subjective sleep quality:

The Pittsburgh Sleep Quality Index (PSQI), as a 19-item self-report questionnaire is used to evaluate the subjective sleep quality in a month (Buysse et al., 1989). The items produce seven component scores. The score of each component ranges from 0 to 3, and the maximum total composite score of the PSQI is 21. The sum of these component scores yields a measure of global subjective sleep quality. The cutoff score of poor sleep quality is 5 or more (Lu et al, 2023). This Chinese version of the PSQI has been validated with adequate reliability in cognitively intact elderly and dementia patients (Blackwell et al., 2014).

2. Attentional function

Complex attention is measured by attention network test (ANT). The ANT paradigm (https://www.sacklerinstitute.org/cornell/assays_and_tools/ant/jin.fan/) is run by E-Prime 3.0 software (Lu et al., 2016). Within ANT paradigm, there are four types of cue: no cue, center cue, double cue, and spatial cue; and three types of flanker: neutral, congruent, and incongruent. The target, a central arrow, could appear above or below the cross-fixation and is surrounded by two flankers on each side.

3. Executive function

Executive function is measured by the category verbal fluency test (CVFT). On each trial, the participants will be asked to overtly generate words in the animal category, fruit category and vegetable category as many as possible within 60 seconds. The total number of correct words is used to measure executive function (Lu et al., 2019).

Secondary outcomes

1. Objective assessment of circadian rhythms

   Actigraphic records are used to quantify sleep-wake cycle and estimate the objective sleep efficiency (Luik et al., 2015). The actigraphy is about the size of a wristwatch and is usually worn on the wrist continuously for multiple days and nights, which can

be an objective measurement of sleep quality and sleep-wake cycle (i.e., circadian rhythms) in the participants.

2. Global cognition is measured by Montreal Cognitive Assessment Hong Kong version (HK MoCA), which is validated global assessment sensitive to detect early cognitive dysfunction in neurocognitive disorder (Lu et al., 2019).

Statistical analysis

The data analyst will be blinded to the grouping of participants. Analyses will be on an intention-to-treat basis. Linear mixed models will be used to assess the differences between conditions on the primary and secondary outcome measures at each time point. This statistical method will facilitate the inclusion of participants with missing data. Treatment, time points, and their interactions will be modelled as the fixed effects. Participants will be modelled as the random effects at time points. Pre-treatment sleep quality, cognitive performance, and the function of glymphatic system will be compared between the randomized groups. Score changes of sleep quality and cognitive functions from baseline to follow-up points across the randomized groups will be tested with occasions (time points) at level one and participants at level two. Covariates identified from baseline differences will be entered in the regression model. Secondary analyses of group differences in outcome of domain-specific functions, and the associations between the changes of PSQI with cognitive functions and the function of glymphatic system will be performed. We will also monitor the incidence of adverse events and characteristics of program adherence. Statistical significance will be set at 2-sided $p < 0.05$. Computations will be performed using R Studio (version 1.1.456).

Sample size calculation

The sample size is considered 15 mild VCI patients in each study arm. This sample size is appropriate for the primary and secondary study objectives. Aiming for the treatment to be acceptable by 70% of the participants with 20% precision (i.e., at least 50% would recommend the treatment), 13 participants are required in the treatment arm. To evaluate the potential efficacy of the treatments as compared with the control and assuming a medium standardised effect size (0.5), 11 participants are required in each group with 80%

on-sided CI approach which is suggested for pilot trials (Cocks & Torgerson, 2013). To account for the follow-up rate of 10%, the total sample size is calculated as 45.

Ethical considerations

Ethics standards will be strictly followed by providing informed consent and respecting anonymity, privacy and confidentiality. Participants will be recruited if the participants are considered mentally fit to sign consent. No personal identity, including name, birth date, mobile numbers, will be revealed in any reports or publications. Participants can withdraw anytime without interference from any future service use. For those who have any medical concerns during the study, they will be advised to seek help from clinical doctors.

Ethics approval from the Clinical Research Ethics Committee (The Joint CUHK-NTEC CREC) will be obtained before commencement of the study. The protocol will also be registered with the Clinical Trials Registry (https://clinicaltrials.gov/). The reporting of trial will follow requirements of major international journals. The study will comply with the Declaration of Helsinki and the Good Clinical Practice (GCP) guidelines of the International Conference on Harmonisation (ICH) of technical requirements for registration of pharmaceuticals for human use (ICH-GCP).

References

Blackwell, T., Yaffe, K., Laffan, A., Ancoli-Israel, S., Redline, S., Ensrud, K. E., & Stone, K. L. (2014). Associations of objectively and subjectively measured sleep quality with subsequent cognitive decline in older community-dwelling men: the MrOS sleep study. Sleep, 37(4), 655-663.

Buysse, D. J., Reynolds III, C. F., Monk, T. H., Berman, S. R., & Kupfer, D. J. (1989). The Pittsburgh Sleep Quality Index: a new instrument for psychiatric practice and research. Psychiatry research, 28(2), 193-213.

Cocks, K., & Torgerson, D. J. (2013). Sample size calculations for pilot randomized trials: a confidence interval approach. Journal of clinical epidemiology, 66(2), 197-201.

Garbarino, S., Lanteri, P., Bragazzi, N. L., Magnavita, N., & Scoditti, E. (2021). Role of sleep deprivation in immune-related disease risk and outcomes. Communications biology, 4(1), 1-17.

Hablitz, L. M., & Nedergaard, M. (2021). The glymphatic system: a novel component of fundamental neurobiology. Journal of Neuroscience, 41(37), 7698-7711.

Lai, M., Gao, Y., Lu, L., Huang, X., Gong, Q., Li, J., & Jiang, P. (2023). Functional connectivity of the left inferior parietal lobule mediates the impact of anxiety and depression symptoms on sleep quality in healthy adults. Cerebral Cortex, 33(17), 9908-9916.

Li, Y., Sahakian, B. J., Kang, J., Langley, C., Zhang, W., Xie, C., & Feng, J. (2022). The brain structure and genetic mechanisms underlying the nonlinear association between sleep duration, cognition and mental health. Nature Aging, 2(5), 425-437.

Liang, T., Chang, F., Huang, Z., Peng, D., Zhou, X., & Liu, W. (2023). Evaluation of glymphatic system activity by diffusion tensor image analysis along the perivascular space (DTI-ALPS) in dementia patients. The British Journal of Radiology, 96(1146), 20220315.

Lu, H., Chan, S. S. M., Chan, W. C., Lin, C., Cheng, C. P. W., & Linda Chiu Wa, L. (2019). Randomized controlled trial of TDCS on cognition in 201 seniors with mild neurocognitive disorder. Annals of clinical and translational neurology, 6(10), 1938-1948.

Lu, H., Fung, A. W., Chan, S. S., & Lam, L. C. (2016). Disturbance of attention network functions in Chinese healthy older adults: an intra-individual perspective. International Psychogeriatrics, 28(2), 291-301.

Lu, H., Ma, S. L., Chan, S. S. M., & Lam, L. C. W. (2016). The effects of apolipoprotein ε 4 on aging brain in cognitively normal Chinese elderly: a surface-based morphometry study. International Psychogeriatrics, 28(9), 1503-1511.

Lu, H., Ni, X., Fung, A. W., & Lam, L. C. (2018). Mapping the proxies of memory and learning function in senior adults with high-performing, normal aging and neurocognitive disorders. Journal of Alzheimer's Disease, 64(3), 815-826.

Lu, H., Li, J., Yang, N. S., Lam, L. C. W., Ma, S. L., Wing, Y. K., & Zhang, L. (2023). Using gamma-band transcranial alternating current stimulation (tACS) to improve sleep quality and cognition in patients with mild neurocognitive disorders due to Alzheimer's disease: A study protocol for a randomized controlled trial. PLoS One, 18(8), e0289591.

Lu, Y., Li, G., Ferrari, P., Freisling, H., Qiao, Y., Wu, L., & Ke, C. (2022). Associations of handgrip strength with morbidity and all-cause mortality of cardiometabolic multimorbidity. BMC medicine, 20(1), 1-11.

Luik, A. I., Zuurbier, L. A., Hofman, A., Van Someren, E. J., Ikram, M. A., & Tiemeier, H. (2015). Associations of the 24-h activity rhythm and sleep with cognition: a population-based study of middle-aged and elderly persons. Sleep medicine, 16(7), 850-855.

Murphy, M., Riedner, B. A., Huber, R., Massimini, M., Ferrarelli, F., & Tononi, G. (2009). Source modeling sleep slow waves. Proceedings of the National Academy of Sciences, 106(5), 1608-1613.

Niethard, N., Ngo, H. V. V., Ehrlich, I., & Born, J. (2018). Cortical circuit activity underlying sleep slow oscillations and spindles. Proceedings of the National Academy of Sciences, 115(39), E9220-E9229.

Paller, K. A., Creery, J. D., & Schechtman, E. (2021). Memory and Sleep: How Sleep Cognition Can Change the Waking Mind for the Better. Annual review of psychology, 72, 123-150.

Peter-Derex, L., Yammine, P., Bastuji, H., & Croisile, B. (2015). Sleep and Alzheimer's disease. Sleep medicine reviews, 19, 29-38.

Potok, W., van der Groen, O., Bächinger, M., Edwards, D., & Wenderoth, N. (2022). Transcranial Random Noise Stimulation Modulates Neural Processing of Sensory and Motor Circuits, from Potential Cellular Mechanisms to Behavior: A Scoping Review. eNeuro, 9(1), ENEURO-0248.

Saleh, A., Schroeter, M., Jonkmanns, C., Hartung, H. P., MoÈdder, U., & Jander, S. (2004). In vivo MRI of brain inflammation in human ischaemic stroke. Brain, 127(7), 1670-1677.

Svensson, T., Saito, E., Svensson, A. K., Melander, O., Orho-Melander, M., Mimura, M., & Inoue, M. (2021). Association of sleep duration with all-and major-cause mortality among adults in Japan, China, Singapore, and Korea. JAMA network open, 4(9), e2122837-e2122837.

van der Groen, O., Potok, W., Wenderoth, N., Edwards, G., Mattingley, J. B., & Edwards, D. (2022). Using noise for the better: the effects of transcranial random noise stimulation on the brain and behavior. Neuroscience & Biobehavioral Reviews, 104702.
